# Objectives, design and enrollment results from the Infant Susceptibility to Pulmonary Infections and Asthma Following RSV Exposure Study (INSPIRE)

**DOI:** 10.1186/s12890-015-0040-0

**Published:** 2015-04-30

**Authors:** Emma K Larkin, Tebeb Gebretsadik, Martin L Moore, Larry J Anderson, William D Dupont, James D Chappell, Patricia A Minton, R Stokes Peebles, Paul E Moore, Robert S Valet, Donald H Arnold, Christian Rosas-Salazar, Suman R Das, Fernando P Polack, Tina V Hartert, On Behalf of the INSPIRE Study

**Affiliations:** Department of Medicine, Vanderbilt University Medical Center, Nashville, TN USA; Department of Biostatistics, Vanderbilt University Medical Center, Nashville, TN USA; Department of Pediatrics, Emory University School of Medicine, Atlanta, GA USA; Department of Pathology, Vanderbilt University Medical Center, Nashville, TN USA; Department of Pediatrics, Vanderbilt University Medical Center, Nashville, TN USA; Virology Department, J. Craig Venter Institute, Rockville, MD USA

**Keywords:** Infants, Bronchiolitis, Respiratory Syncytial Virus, Asthma, Rhinovirus, Allergic Rhinitis, Wheezing, Respiratory Tract Infections

## Abstract

**Background:**

Respiratory syncytial virus (RSV) lower respiratory tract infection (LRI) during infancy has been consistently associated with an increased risk of childhood asthma. In addition, evidence supports that this relationship is causal. However, the mechanisms through which RSV contributes to asthma development are not understood. The INSPIRE (Infant Susceptibility to Pulmonary Infections and Asthma Following RSV Exposure) study objectives are to: 1) characterize the host phenotypic response to RSV infection in infancy and the risk of recurrent wheeze and asthma, 2) identify the immune response and lung injury patterns of RSV infection that are associated with the development of early childhood wheezing illness and asthma, and 3) determine the contribution of specific RSV strains to early childhood wheezing and asthma development. This article describes the INSPIRE study, including study aims, design, recruitment results, and enrolled population characteristics.

**Methods/design:**

The cohort is a population based longitudinal birth cohort of term healthy infants enrolled during the first months of life over a two year period. Respiratory infection surveillance was conducted from November to March of the first year of life, through surveys administered every two weeks. In-person illness visits were conducted if infants met pre-specified criteria for a respiratory illness visit. Infants will be followed annually to ages 3-4 years for assessment of the primary endpoint: wheezing illness. Nasal, urine, stool and blood samples were collected at various time points throughout the study for measurements of host and viral factors that predict wheezing illness. Nested case-control studies will additionally be used to address other primary and secondary hypotheses.

**Discussion:**

In the INSPIRE study, 1952 infants (48% female) were enrolled during the two enrollment years and follow-up will continue through 2016. The mean age of enrollment was 60 days. During winter viral season, more than 14,000 surveillance surveys were carried out resulting in 2,103 respiratory illness visits on 1189 infants. First year follow-up has been completed on over 95% percent of participants from the first year of enrollment.

With ongoing follow-up for wheezing and childhood asthma outcomes, the INSPIRE study will advance our understanding of the complex causal relationship between RSV infection and early childhood wheezing and asthma.

## Background

Prospective epidemiologic studies have consistently demonstrated that infants with respiratory syncytial virus (RSV) lower respiratory tract infections (LRIs) have an increased risk of early childhood wheezing and asthma [1-5]. There is also evidence from animal [6-10], human [8,11], and clinical studies [12,13] that mild RSV infections may reduce the risk of asthma development, presumptively by modulating pathogenic immunity through early activation and expansion of T regulatory cells. Intervention trials in humans demonstrating that preventing infant RSV illness prevents recurrent wheezing, and mechanistic studies in animal models provide proof of concept and plausible mechanisms through which RSV could cause asthma [14-17]. Additional research demonstrates that the strain of RSV is associated with the clinical severity of acute infection [18-20]. Thus, RSV may be an important organism that modifies the developing immune system, differently, depending on the severity or host response to the virus. This is important, as RSV prevention or modulating host response to viral infection could result in a new target for primary asthma prevention. The INSPIRE (Infant Susceptibility to Pulmonary Infections and Asthma Following RSV Exposure) study builds upon the known links between RSV LRI and wheezing illness to address unanswered questions about the role of mild RSV infection in decreasing the risk of childhood wheezing illness and host and viral characteristics associated with wheezing or asthma following infant RSV infection.

**Figure 1:**
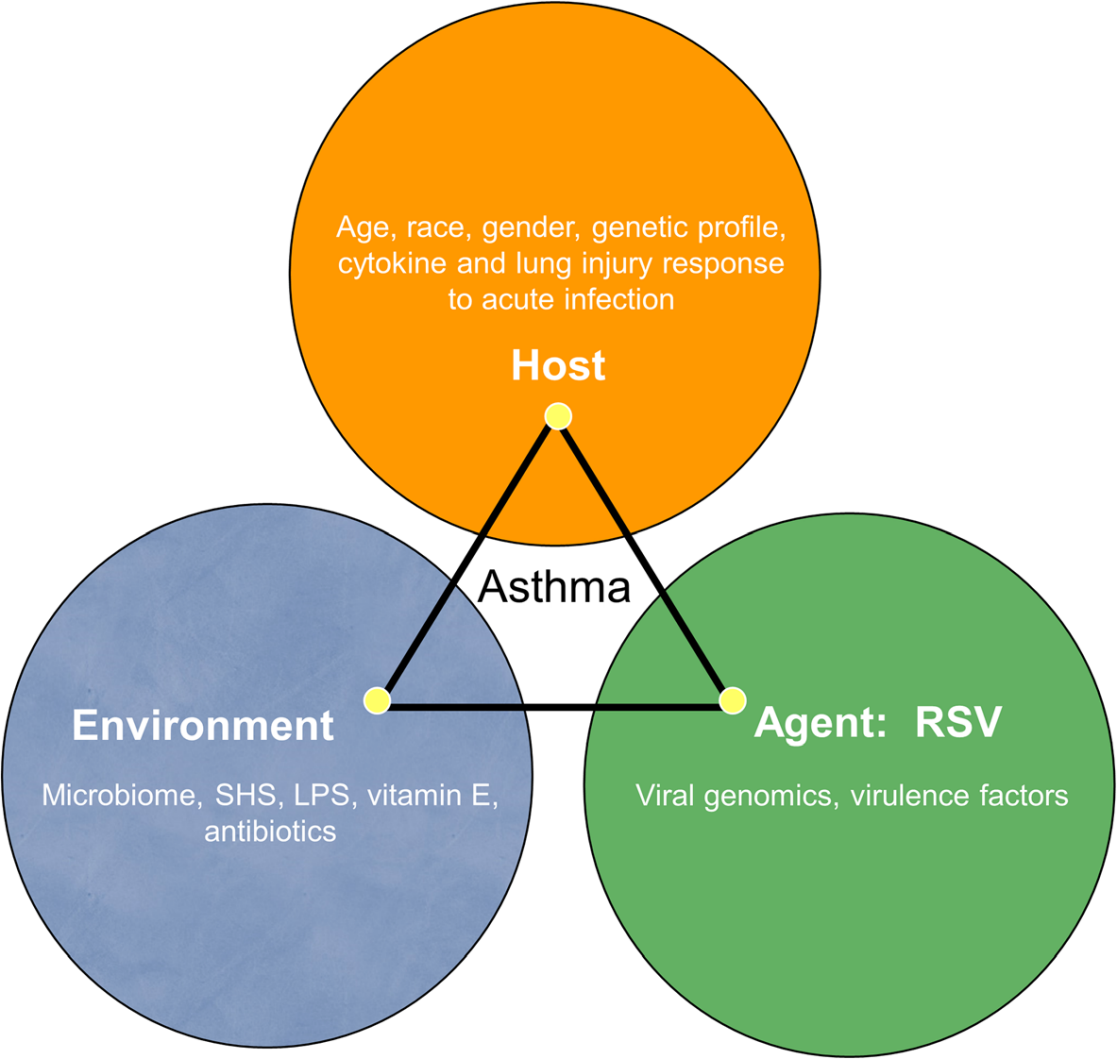
Hypothesis Testing: The epidemiologic triangle.

Because we cannot yet predict which infants with RSV LRI will ultimately develop childhood asthma and wheezing illnesses, or who might most benefit from preventing early viral infection [1,21,22], the INSPIRE study cohort was designed to help understand how the complete spectrum (mild to severe) of RSV illnesses contributes to early childhood wheezing and asthma. In particular, because there is a paucity of studies on outpatient RSV, our study captures mild RSV infections. This longitudinal cohort was specifically designed to 1) establish the relationship between the host phenotypic response to RSV infection in the first 6 months of life and the risk of recurrent wheeze and asthma; 2) identify the host immune response determinants of the RSV infection phenotype on the development of early childhood wheezing and asthma following RSV infection; and 3) determine the contribution of specific RSV strains to early childhood wheezing and asthma development. Figure 1 depicts the conceptual framework that the study is built upon.

This paper details the study aims, procedures, and recruitment results from the INSPIRE study, a unique birth cohort of over 1,900 term, healthy infants followed longitudinally from infancy through early childhood for the outcomes of wheezing illnesses and asthma, with detailed characterization of respiratory illness during infancy during RSV season. The study will identify host, viral and environmental factors associated with RSV infection in infancy and the development of childhood asthma.

## Methods

### Primary objectives

The study was designed to test two primary hypotheses: 1) the severity of RSV infection during infancy is associated with recurrent wheezing and early childhood asthma in a severity-dependent manner and; 2) indices of host immune response and lung injury associated with infant RSV infection will differ among infants who do and do not develop recurrent wheezing and early childhood asthma.

### Secondary objectives

The secondary objectives are to: 1) test the hypothesis that specific RSV strains will be associated with differential LRI severity, recurrent wheezing and early childhood asthma; 2) determine the association between all viral infections (URIs only (mild) any LRI (severe)) and allergic rhinitis (AR); 3) test the interactions between allergic rhinitis/laboratory atopy and smoking, family history of asthma, infant age at RSV infection with respiratory infection severity; 4) assess the relationship between LRI severity and allergic rhinitis within the LRI subgroup; 5) determine the association of RSV infection severity with recurrent wheezing and wheezing illness severity; 6) test the hypothesis that there are RSV strain differences in the pattern of cytokines in acute infection; 7) longitudinally characterize the airway microbiome in early infancy, during infant RSV acute respiratory illness (ARI), and at 2-3 years of age on a subset of children (n = 100); 8) determine whether the specific patterns of airway microbiome diversity in early infancy (prior to RSV ARI), during infant RSV ARI, and/or the change in pattern between health and RSV ARI are associated with respiratory outcomes of severity of RSV ARI in infancy and subsequent development of wheezing illness in early childhood; and 9) determine whether specific patterns of airway microbial diversity in early infancy (prior to RSV ARI), during infant RSV ARI, and/or the change in pattern between health and RSV ARI are associated with patterns T cell responses and innate cellular responses to RSV stimulation at 2-3 years on a subset of infants.

### Study design

To address primary hypothesis 1, we designed a longitudinal observational birth cohort study of infants (the INSPIRE cohort), enrolled over a two year period, which included respiratory illness surveillance from November 1 to March 31 during the infant’s first year of life, and annual follow-up to the third or fourth birthday. Primary hypothesis 2 will be addressed through the use of a nested case-control study from participants within the cohort.

### Study population and subject recruitment

We enrolled and are longitudinally following 1952 term, otherwise healthy infants from pediatric practices located in Middle Tennessee, or self-referral through research announcements. The recruitment area encompasses urban, suburban and rural areas. Infants meeting eligibility criteria outlined in Table 1 were approached at time of a pediatric visit to a participating practice. By design, infants whose birthdays fell between June 1^st^ and December 31^st^ were targeted for enrollment so that infants were on average younger than 6 months of age during winter virus season, when RSV infection is likely to both represent the first and most severe infection. To meet recruitment milestones and assure sufficient spectrum of disease severity during the 2 recruitment seasons, infants were also enrolled during acute illnesses. The protocol and informed consent documents were approved by the Institutional Review Board at Vanderbilt University Medical Center. One parent of each participant provided written informed consent for participation in this study. The informed consent document explained study procedures, use of data of and biospecimens for future studies, including a rider for genetic studies.

**Table 1:** **Inclusion and Exclusion Criteria for Enrollment in INSPIRE Study**

**Inclusion criteria:**	**Exclusion criteria:**
Singleton birth to a mother 18 years or older.	Need for mechanical ventilation prior to enrollment
Born at ≥ 37 weeks gestation	Significant cardiopulmonary disease
Birth weight ≥ 2250 g (5 lbs.)	Bronchopulmonary dysplasia
Born June 1 – December 31 during enrollment years: 2012, 2013	Cystic fibrosis
Guardian/parent able to understand and provide informed consent	Immunodeficiency (including maternal HIV)
No intent to relocate from Nashville and surrounding area within 5 years	Neurological disease
	Any other health condition that may jeopardize the integrity of data to be collected during the study

### Study visits and procedures

#### Enrollment visit

At the baseline visit, trained study staff administered a baseline questionnaire. The components of the study visits are given in Table 2. The questionnaire included demographic characteristics, infant medical history, family history, secondhand smoke exposure, daycare attendance, growth trajectory and other factors known to be associated with childhood asthma and allergy development. Multiple bio-specimens were collected for different purposes at each time point and are described in Table 3. A urine sample was collected at enrollment, through use of an external bag, for markers of inflammation and lung injury. In year 2, infant stool samples and nasal samples were also collected at enrollment. The infant nasal samples at enrollment were collected by insertion of filter paper (Leukosorb™; Pall Corporation, Port Washington, NY) into each nostril, as described previously [23,24]. A maternal finger stick was also collected in year 2 to bank maternal blood samples for other assays, such as vitamin E isoforms.

**Table 2:** **INSPIRE Main Study Schedule**

**Time Point**	**First year enrollment timeline**	**Second year enrollment timeline**	**Visit Type**	**Survey**	**Nasal**	**Urine**	**Fecal**	**LPS Wipe**	**Blood**	**Allergy**
Enrollment Visit	June 2012 –Mar 2013	June 2013 – Mar 2014	Baseline	X	X*	X	X*		X maternal	
Winter Virus Season	Nov 2012 –Mar 2013	Nov 2013 - Mar 2014	Biweekly Contact	X						
			Multiple Respiratory Illness visits	X	X	X				
Well Child Visits	June – Dec 2013	June – Dec 2014	1^st^ Annual Visit	X				X	X	
	June – Dec 2014	June – Dec 2015	2^nd^ Annual Visit	X						
		June 2015 –June 2016**	2-3 Year Sub-Study (n = 100)**	X	X		X		X PBMC**	
	June – Dec 2015	June – Dec 2016	3^rd^ Annual Visit	X						X
	June – Dec 2016		4^th^ Annual Visit	X						

*included in second year cohort enrollment only.

**Sub-study of microbiome on subset of children; PBMC = Peripheral Blood Mononuclear Cells.

Nasal = Filter paper at baseline and nasal wash at time of respiratory illness.

Blood = Maternal finger stick at baseline and infant venipuncture or finger/toe stick at 1 year.

Allergy = Skin prick testing for aeroallergens or venipuncture for serum allergen-specific IgE.

**Table 3:** **Specimen Repository for INSPIRE Study**

**Time Point and Sample Type**		***A priori*** **testing planned**
**Baseline:**		
	Infant urine	Markers of inflammation and lung Injury
	Infant nasal filter paper*	Respiratory microbiome, cytokines
	Infant stool sample*	Gut microbiome
	Maternal plasma*	Vitamin E levels
**Respiratory Illness:**		
	Infant urine	Markers of inflammation and lung injury
	Infant nasal wash	PCR identification of RSV or RV; cytokines and biomarkers of immune function; microbiome. RSV positive samples will be sequenced.
**One Year:**		
	Infant blood	RSV serology, DNA banking
	Home doorframe wipe	Endotoxin/Lipopolysaccharide
**Two Year Sub-Study:**		
Child peripheral blood mononuclear cells		Immune response to RSV stimulation
Child nasal wash		Nasal microbiome
Child stool sample		Gut microbiome
**Three Year:**		
Child blood		Serum allergen-specific IgE measurements; if skin prick testing not possible

Bold indicates study time point.

*Collected only in second year of enrollment.

#### Respiratory illness surveillance

Respiratory illness surveillance was conducted during the winter virus season (November to March) of each infant’s first year of life in order to capture and characterize infant acute respiratory illnesses during RSV season. Respiratory illness surveillance had three components: first, parents were instructed to call the research staff between November and March, at first onset of respiratory symptoms which included any one of the following: runny nose, congestion, wheezing, fever and/or cough at which time the screening respiratory illness survey was completed. Second, all enrolled infants who were seen for an acute visit at their pediatric practice were approached and administered the respiratory illness survey. Third, every two weeks, from November through March, parents received regular contact by an e-mail secure survey or telephone call to complete the respiratory illness survey. The respiratory illness survey is a series of questions about respiratory symptoms and time of onset.

#### Acute respiratory illness visits

Infants met our case definition of a respiratory illness visit based on responses to the respiratory illness surveillance questionnaires: a parent indicates *ONE* of the following major symptoms or diagnoses: wheezing, difficulty breathing, or told that your baby had a positive RSV test *OR ANY TWO* of the following minor symptoms or diagnoses: fever, runny nose/congestion/snotty nose, cough, ear infection (otitis media), or hoarse cry. Onset of symptoms must have been in the prior week (the past 5 days for the infants enrolled in year 1 of the study, and the prior 7 days for infants enrolled in year 2 of the study). This change in symptom/diagnosis window was made to accommodate missing respiratory visits because of no staffing on weekends.

If the infant met pre-specified criteria for a respiratory illness visit, an in-person visit was performed either in the clinic, the pediatric clinical research center, or the infant’s home. The visit included a brief physical exam with elements that measure the severity of respiratory infection: respiratory rate, oxygen saturation/heart rate, wheezing, and chest retractions. Chart review was used to supplement the research exam with information about doctor diagnosis, when a healthcare visit occurred. The respiratory symptom assessment detailed the symptoms present and the date of onset. In addition, a nasal wash and urine sample were collected at each respiratory illness visit. A nasal wash was conducted using 5 mL of sterile saline solution and immediately placed at 4 degrees Celsius. The nasal washes were aliquoted and snap frozen at -80 degrees within 24 hours of collection.

#### 1-year study visit

The one year follow-up visit was conducted between 9 – 15 months of age and included questions about respiratory health and exposures, and a venipuncture for RSV serology. If the caregiver refused venipuncture or blood was not able to be drawn, research staff collected 250 μL of blood from a toe or finger stick. If consent was given for genetic studies, a buffy coat sample was banked for future DNA extraction. Participants were also asked to collect household dust samples by wiping the top of the doorframe of the room where the infant sleeps with 2 wipes (TefTex™, Dalla Lana School of Public Health, University of Toronto, Toronto, Canada) and returning by mail [25]. These samples are to be used for endotoxin/lipopolysaccharide measurements [25,26].

#### Annual visits

Annual follow-up study contact is ongoing and will take place at year 2, 3 and 4. Year 2 and 4 follow-up is by telephone survey and includes the International Study of Asthma and Allergy in Children (ISAAC) [27] and other allergic disease and respiratory disease questionnaires. The third year visit is in-person and includes the above components, as well as skin prick testing to aeroallergens common to the region, or if skin testing cannot be performed, a blood draw for measurement of specific IgE to the same aeroallergens.

### Study endpoints

The co-primary study endpoints are both recurrent wheezing and early childhood asthma. Recurrent wheezing is defined between ages 1-3 years, as two or more wheezing episodes at any time during that period. Early childhood asthma will be determined at age four using elements of the ISAAC questionnaire. Children who meet both of the following criteria at age four will be classified as definite early childhood asthmatics: (1) 12-month prevalence of asthma symptoms or exercise-induced wheeze or dry cough at night, AND (2) physician diagnosis by parental report or use/prescription of asthma medications [27-36]. Children who meet just one of the two criteria will be considered to have probable early childhood asthma.

### Secondary endpoints

Respiratory illness severity at time of illness will be measured by an ordinal scale based on wheezing, respiratory rate, retractions, and oxygen saturation or heart rate, modified from scores derived for LRI [37]. Also, a dichotomous outcome, LRI versus URI will be used to measure severity. Allergic rhinitis (AR) diagnosis will also be made using elements of the ISAAC questionnaire. Children will be considered to have *definite allergic rhinitis* (AR) if each of three conditions are present at age four: (1) a history of nasal congestion, runny nose, itchy watery eyes, sneezing, or blocked nose; *AND* (2) substantial variability in symptoms over time or seasonality; *AND* (3) diagnosed as having AR by a physician or on medications for AR. Patients will be considered to have *probable AR* if they report symptoms as outlined above in (1) and (2) but have not been diagnosed or treated by a physician. Atopic dermatitis diagnosis will be confirmed through the ISAAC core questions on atopic dermatitis, which are based on a list of major and minor criteria proposed by Hanifin and Rajka in the 1970s [32,38,39]. Patients will be classified as definite atopic dermatitis if they report ever having an itchy rash that comes and goes for at least six months, AND being diagnosed with eczema by physician documentation. They will be classified as probable atopic dermatitis if they report one of the two above criteria. Atopy will be assessed by either a positive skin prick allergen test for aeroallergens (preferred) or specific IgE from blood test at age 3 years: 1) Positive skin prick testing is defined by a wheal (center raised part) of 3 mm or greater to one of the aeroallergens (when histamine control is positive and the saline control is negative [non-reactive], indicating that the patient is not dermatographic [i.e. reacting to irritation and not to specific allergens]), or 2) laboratory atopy will be defined as specific IgE > 0.1 PAU/L to at least one common aeroallergen performed on blood specimens collected at 3-years. Additional secondary endpoints will be defined by breaking down co-primary recurrent wheezing outcome into well-accepted asthma phenotypes [40], including: 1) transient early wheezing will be defined as wheezing episodes during the first three years of life [41]. 2) persistent wheezing will be defined as wheezing during the first three years and beyond. 3) Late onset wheezing will be defined as wheezing not present in the first three years, but with symptoms beginning between three and four years.

### Primary exposure

Singleplex reverse-transcription real-time PCR assays for RSV, human rhinovirus, human enterovirus, and human ribonuclease P (RNAseP) in nasal washes were performed according to published protocols [42,43]. RNAseP served as an endogenous control for specimen quality, and specimens failing to demonstrate an RNAseP signal among multiple individually processed aliquots were deemed indeterminate for viruses not detected in repetitive testing. Efficiency of nucleic acid extraction was monitored through amplification of RNAseP in cultured human-cell lysates incorporated into each round of specimen processing. Results of amplification reactions were validated by expected findings for water blanks, negative controls, and positive controls derived from viral culture lysates (adjusted to concentrations representative of patient specimens) included in each PCR reaction plate. RSV IgG antibodies will be assessed at one year by four enzyme immunoassays (EIA). One EIA using RSV A2 (group A strain) lysate as the antigen, one using RSV B1 (group B strain) lysate, one using an RSV group A G protein peptide (RSV A2 aa 161-190 : NDFHFEVFNF VPCSICSNNP TCWAICKRIP), and one using an RSV group B G protein peptide (RSV B1 aa 161-190 : DDYHFEVFNF VPCSICGNNQ LCKSICKTIP). RSV serology at year one, coupled with viral identification of RSV by PCR at time of respiratory illness, will be used to determine whether infants were exposed to RSV.

### Biomarkers analyzed during acute respiratory illness

Additional biomarkers analyzed during acute respiratory illness will also serve as primary endpoints for analyses. Lung injury and host immune response will be measured in urine samples collected during the acute respiratory illness and include cysteinyl leukotrienes, CC-16, KL-6, IL-18, TNF-α, HNE, prostacyclin, and isoprostanes. Immune function biomarkers assayed from nasal washes collected during acute respiratory illness include: VEGF, G-CSF, EGF, FGF, eotaxin, eotaxin-2, eotaxin-3, MCP-2, BCA-1, MCP-4, I-309, IL-16, TARC, X6CKine, LIF, TPO, SCF, TSLP, IL-33, IL-20, IL-21, IL-23, TRAIL, CTACK, SDF-1a-b, ENA-78, MIP-1d, IL-28A, IL-1B, IL-10, IL-13, IL-6, IL-12, RANTES, IL-17, MIP-1a, GM-CSF, MIP-1B, MCP-1, IL-15, IL-5, IFN-y, IFN-a, IL-1RA, TNF-a, IL-2, IL-7, IP-10, IL-2R, MIG, IL-4, IL-8, HGF and Muc5ac.

### Planned statistical analyses

Descriptive analysis of infant demographic, clinical and biological measurements will be performed using means and standard deviations or medians and inter-quartile ranges for continuous variables, and proportions for categorical variables.

### Cohort study

Our first hypothesis examines whether the severity of RSV infection is associated with risk of early childhood wheezing or early childhood asthma. RSV exposure is determined by confirmed RSV illness by PCR, and/or positive 1-year RSV serology. The co-primary endpoints will be recurrent wheezing and early childhood asthma as described previously. Logistic regression will be used to estimate the odds ratio (OR) and 95% confidence intervals [44] of developing childhood asthma or recurrent wheezing in children with severe or mild RSV infection relative to those who were not infected in infancy. Both crude ORs and ORs adjusted for confounding variables will be derived. Confounding variables for our multiple logistic regression models will include maternal age, infant age at first RSV infection, gestational age, secondhand smoke (SHS), infant sex, and family history of allergic diseases. Other confounding variables to be considered include further detail on home environment, including infant diet, use of acetaminophen or NSAIDs, study year, gender, and family history of asthma. A priori interaction terms will be included in our models and we will examine their effect on the change of model deviance. Additional analyses will be conducted to assess model fit. These will include scatter plots of squared standardized Pearson residuals against the estimated asthma probability for patients with each covariate pattern. Analyses will be conducted using SAS (SAS Institute Cary, NC) and R (http://www.r-project.org).

### Nested case-control study

To test our hypothesis that measures of host immune response and lung injury will differ between infants with RSV illness who do and do not develop recurrent wheezing or early childhood asthma, we will conduct nested case-control studies of incident early childhood asthma cases following infant RSV infection to matched RSV-infected children who do not develop recurrent wheezing or asthma. Two controls will be selected for each case as follows: First, we will fit a logistic regression model in which asthma outcome is regressed against these matching covariates. The coefficients from this model will be used as weights to create an asthma risk score for each subject, which is the weighted sum of these risk covariates. Controls will then be frequency matched to cases based on their asthma risk score. This risk score will be derived from one of our prior analyses of an administrative data set that used a regression analysis [45].

### Power calculations

**Figure 2:**
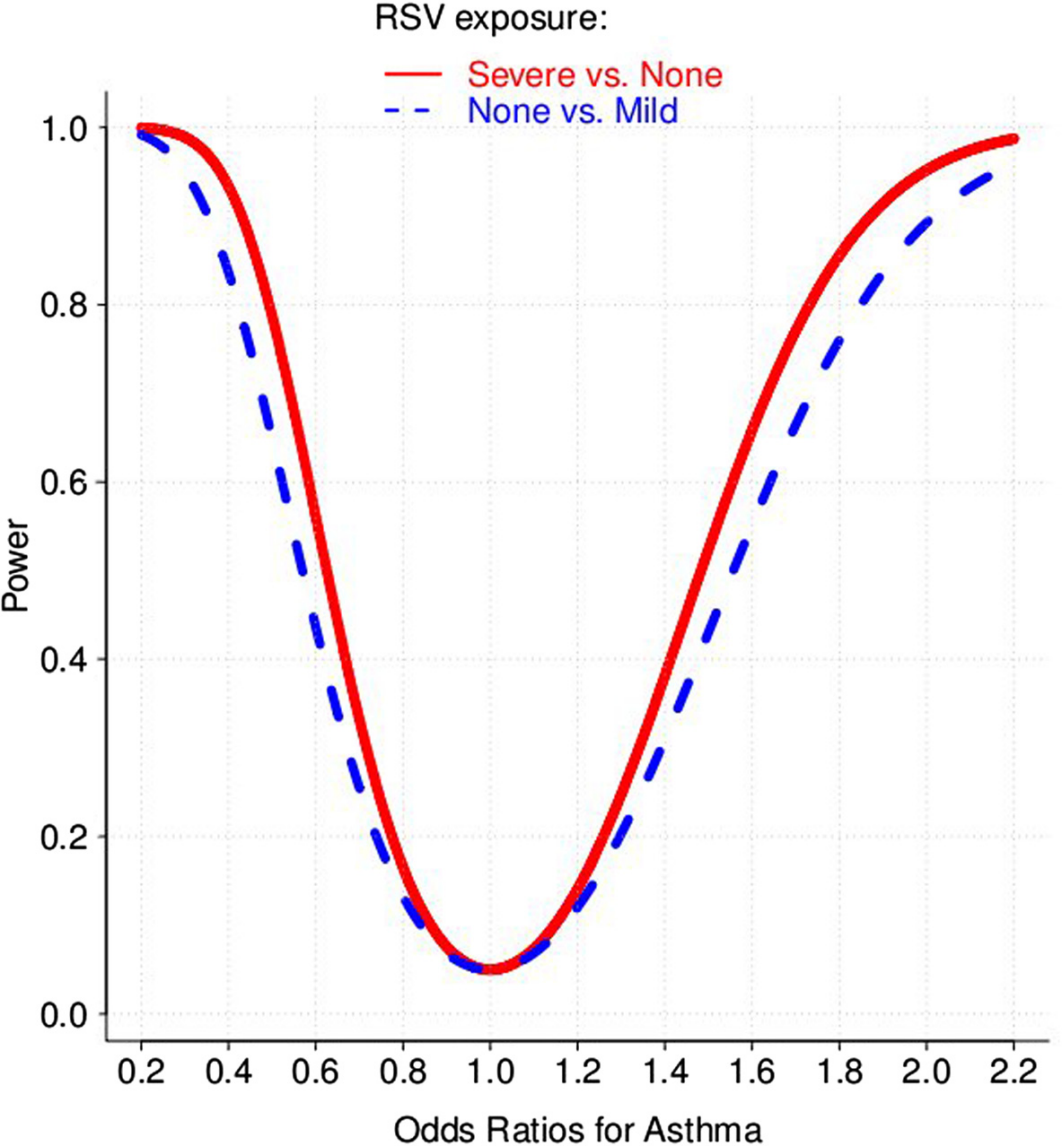
Power for primary hypothesis 1 to assess the association between RSV exposure and asthma based on 1900 infants with 25% attrition (α = 0.05).

Primary Hypothesis 1: Power calculations were performed before the study began. The primary outcome for this study is recurrent wheezing illness at age 3-4, rather than asthma, which requires more time for follow-up than allowed during the grant funding period. Our power calculations were based on a birth cohort of 1500 infants plus a total of 400 infants recruited at the time of RSV infection in infancy for a total of 1900 children. Two thirds of these 400 were assumed to have severe RSV disease necessitating hospitalization while one-third to have mild disease treated on an outpatient basis. We anticipated that 75% of the subjects will be successfully followed up to measure asthma outcomes at 4-6 years of age. We have powered our primary hypothesis with RSV exposure levels of 20% of infants with LRI, 40% URI and 40% with no RSV infection in their first year of life. We further assumed a conservative estimate of 11% infants who were not exposed to RSV to develop asthma at ages 4-6 years. We performed power calculations based on a 2-sided test of the null hypothesis that the asthma prevalence was not affected by infant RSV exposure, with a Type I error probability of α = 0.05. In Figure 2, the red curve shows the power for different asthma odds ratios for infants with RSV LRI relative to those with no infection. We expect that the prevalence of asthma among infants with URI (mild) will be 7% and the dashed blue curve in Figure 2 shows the asthma odds ratios for infants with no exposure vs. mild infection. The prevalence of recurrent wheezing is expected to be much higher than the asthma prevalence shown, thus we expect to have ample power for our analysis. We anticipate that 30% of the entire cohort will have recurrent wheezing at three years of age. This will provide us with considerably more power to detect different odds ratios for wheezing than we will attain for asthma, as shown in Figure 2. These power calculations show we will have at least 80% power to detect recurrent wheezing relative risks greater than 1.65 or less than 0.61 in subgroups of infants defined by RSV exposure and assuming 75% follow up.

Primary Hypothesis 2, Nested Case-Control Study: Preliminary data from previous studies provide estimates of selected biomarkers, including leukotrienes, CC-16, KL-6, IL-18, TNF-α, and HNE. Estimates of minimal detectable differences (two sided α = 0.05) are given in Table 4, for different numbers of asthma cases identified [46-50].

**Table 4:** **Nested case-control study, minimal detectable differences of biomarkers under different hypothetical assumptions with 1:2 case-control matching**

**Biomarkers**	Mean (SD)*	**20% RSV exposed: 83 asthma cases**	**30% RSV exposed: 166 asthma cases**
Urine Leukotrienes	83.8 pg/mg (38.2)	14.4	10.2
KL-6	127.1 U/mL (69.1)	26.2	18.4
Urinary PGI	175 pg/mL (12.1)	4.6	3.2

* Based on references [46-50].

### Recruitment results

#### Screening and enrollment

**Figure 3:**
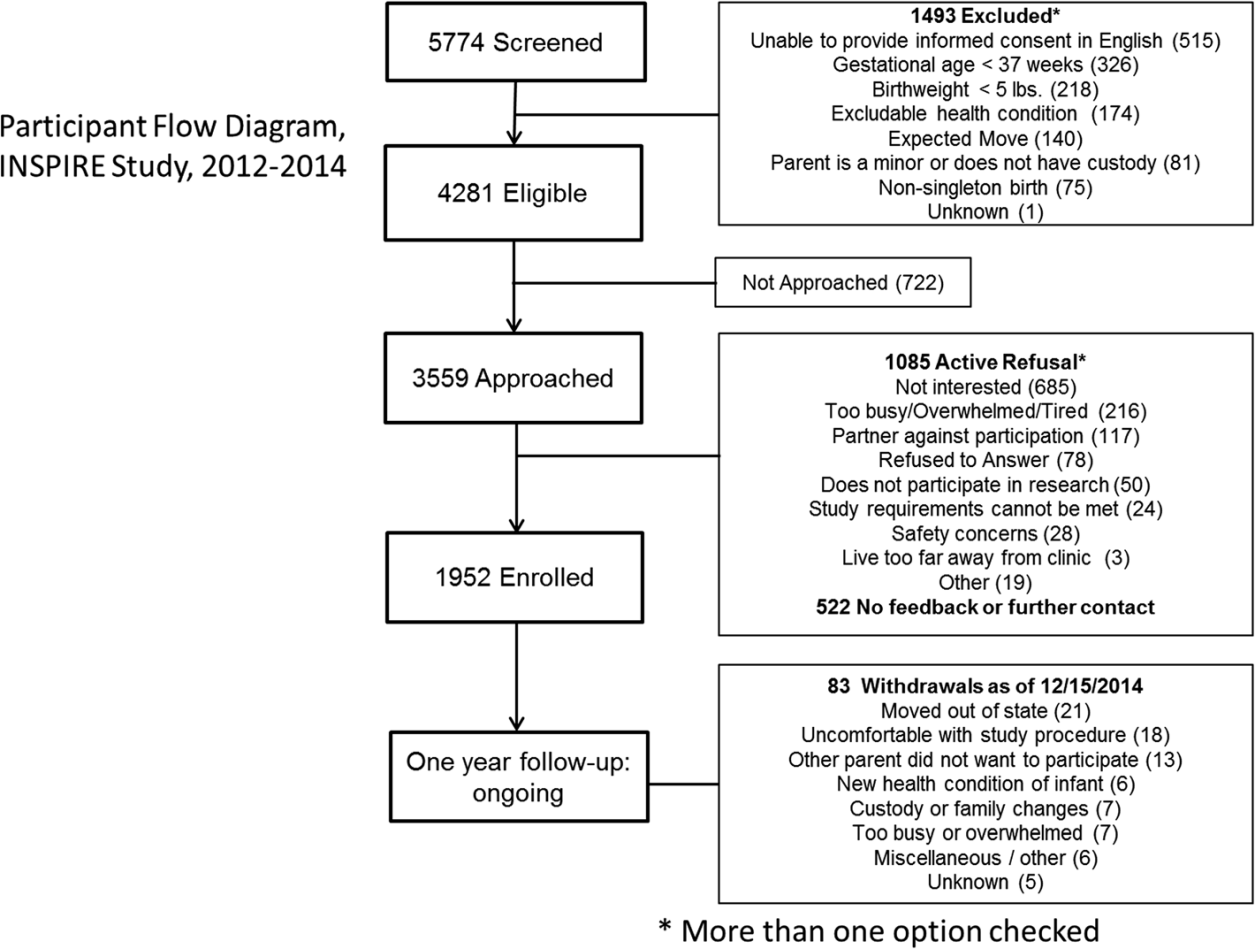
Flowchart of INSPIRE Study, through December 15, 2014.

An overview of the recruitment is presented in Figure 3. 1952 infants were enrolled: 861 infants in the first year and 1091 in the second year. We approached 83% of eligible infants (3559/4381) for enrollment with 55% agreed to participate (1952/3559). The most common reason participants provided for not enrolling was that they were “not interested” (63%; n = 685/1085). Five hundred and twenty-two parents received brochures and study information, but decided not to enroll at the time of visit. In this situation, we were not able to establish contact again, despite a continued presence at their pediatrician’s office and do not know the reason for non-enrollment. At the time of this publication, 82 participants have withdrawn from the study, most commonly due to an unexpected move out of Middle Tennessee (N = 21). The second most common reason was discomfort with the 1-year blood draw (N = 18). For those willing to participate after moving, a blood sample will be collected, processed and shipped, and the annual case report form will be administered by telephone.

Demographic characteristics of the sample are shown in Table 5. Approximately 93% percent of our participants were enrolled from 8 pediatric practices, where staff was located on-site throughout the study. The remaining visits were performed at the Vanderbilt Pediatric Clinical Research Center (PCRC). Eight percent (164/1952) of infants had an acute respiratory infection at the time of enrollment. Among those age-eligible for the 1-year visit, 85% have been completed as of December 15, 2014.

**Table 5:** **Demographic Characteristics of Infants in the INSPIRE Study**

**Baseline demographics**	**N = 1952**
Race, n (%)	
White Non-Hispanic	1269 (65%)
Black Non-Hispanic	345 (18%)
Hispanic	171 (9%)
Multiracial	138 (7%)
Asian	24 (1%)
Other	5 (0%)
Sex, n % Male	1021 (52%)
Mean infant age at enrollment in days, (SD)	59.6 (51.5)
Currently breastfeeding at enrollment, n (%)	1018 (52%)
Mean gestational age in weeks, (SD)	39.1 (1.1)
Mean birth weight in grams (SD)	3425 (515)
Exposure to Prenatal Smoking, n (%)	352 (18%)
Maternal asthma, n (%) enroll	380 (19%)
Married, n (%)	1124 (58%)
Insurance	
Medicaid	1060 (54%)
Private	868 (44%)
Other/Unknown	24 (2%)
Mother employment status, n (%)	
Full Time	862 (44%)
Part Time	403 (21%)
Not Employed	679 (35%)

#### Acute respiratory illness surveillance

Over the two respiratory seasons between November through March, 14,285 respiratory surveillance contacts were completed through e-mail, telephone or in-person on 1,943 individuals (9 individuals never responded). The median time between contacts was 14 days with an interquartile range (IQR) of 12.6-15.6 days. The median number of responses per individual was 8 (IQR: 6-10.5). 5,200 survey respondents reported symptoms within the past 5 days; however, only 2,516 met criteria for an acute respiratory illness visit by either 1 major symptom or 2 minor symptoms.

#### Respiratory illness visits

2,103 respiratory illness visits were completed on 1,189 infants, with 2,036 nasal washes collected and stored. Of these 61% of infants had one or more respiratory illness visits. 682 infants had 1 visit, 314 had 2 visits, 149 had 3 visits and 78 had 4 or more visits. Urine was collected during 1652 (79%) respiratory illness visits. 385 (19%) infant respiratory illness visits were RSV positive based on PCR of nasal washes. Blood was banked for RSV serology testing on 1-year infant samples.

#### Longitudinal follow-up

At the time of this publication, 2-year telephone calls are ongoing for those recruited in the first year of enrollment and 1-year visits are ongoing for infants enrolled in the second year. We have completed approximately 95% of the 1-year follow-up visits for first year of enrollment, after excluding withdrawals (760/805).

## Discussion

Currently, INSPIRE is the largest population based prospective epidemiologic study where the spectrum of severity of RSV infection illnesses are assessed in relationship to recurrent wheezing illnesses and asthma onset. Asthma is a multifactorial disease with onset, course and severity reflecting the combined effects of development-specific exposures *in utero* and post-natally, and the host responses to those exposures. RSV is one of these ubiquitous exposures, which as an infectious agent likely works through differentially modifying the developing immune system and lung, based on specific strains, severity of infection, and host response to infection. The first primary study hypothesis uses the entire cohort to determine the relationship between the spectrum of RSV exposure to severe infection on risk of wheezing illness in childhood. While severe LRI has been linked to development of wheezing or asthma [1-5,51], little is known about the role of mild infection.

The INSPIRE study was designed to address gaps in our knowledge about the role of the host immune response and lung injury to RSV infection in asthma inception, and to understand how viral strain differences contribute to infant morbidity and asthma risk. This will be done through the second study hypothesis that utilizes a nested case-control design within the total cohort. This design will identify host immune and lung injury markers that are differentially associated with outcomes of wheezing illnesses at ages 3-4, and eventually childhood asthma at age 6. Our hypothesis is based on evidence that host responses are associated with increased risk of severe RSV LRI. Among infants with severe RSV infection, TNF-α and eosinophil active cytokines are elevated compared to non-ill infants [52] or mildly ill infants [53,54] and correlate with the severity of illness [55]. After RSV LRI, cysteinyl leukotrienes and IL-17 remain elevated in nasal epithelium [53,56], and IL10 production and genetic variants IL-13, IL-19 and IL-20 are associated with post-bronchial wheeze [57-60]. RSV infection may also cause harmful remodeling of infant lungs [40]. Infant lungs are not fully developed at birth; changes in airway epithelium and alveolarization continue through the first two years of life [61]. Animal studies have shown increased airway hyper-responsiveness and decreased neuronal relaxation after experimental RSV infection [62]. The INSPIRE study will bridge what is known about the immune response at time of infection and extend to 3-4 year outcomes of wheezing illnesses. RSV strains from infants in INSPIRE will be sequenced to determine if strain differences are associated with acute infection morbidity in infancy and later wheezing outcomes.

Prior studies of infant RSV infection have focused almost exclusively on the 3-5% of infants hospitalized with RSV LRI. Most infants, however, have milder RSV infection, and the majority of those who do develop RSV LRI do not develop asthma. While severe LRI has been clearly linked to the development of asthma, an important unanswered question is whether mild RSV infection confers intermediate risk or may actually protect against the inception of childhood asthma. If mild RSV infection primes the immune system or confers a protective effect against future wheezing illnesses, this has important implications for the development of RSV vaccines and RSV immunoprophylaxis. Thus, there are compelling reasons to focus on the development of new approaches to understanding why certain infants are predisposed to developing viral LRI, and which children with RSV LRI develop subsequent childhood asthma.

Strengths of our study include biweekly surveillance to capture the full spectrum of respiratory infections, including even mild infections by regular surveillance intervals, and serologic confirmation of infant infection to identify those who are infected and those who are unexposed. The location of research staff at pediatric clinics allowed frequent contact with participants throughout winter virus season. We also have a large repository of banked urine samples during enrollment and acute illness, 1-year plasma samples, DNA, stool samples, acute illness nasal washes, and baseline enrollment nasal filter paper samples.

This study has several limitations that are important to consider. First, the study sample was not selected from the general population, but instead was recruited from participating pediatric clinics in the city of Nashville and surrounding counties; however, we attempted enrollment of all births at these clinics meeting criteria. Our study cohort demographics represent a varied population demographic that reflects the diversity of the community from which the cohort is recruited. Surveillance for respiratory illnesses occurred during a full 5-month period. However, surveillance was by biweekly surveys or self-reporting, and illnesses may have been missed or not reported. In addition, the respiratory illness visits may have occurred up to 7 days after onset of symptoms, thus characterization of the illness severity at the visit may not reflect the time when the illness was most severe. Although we decided to include an enrichment sample of infants with acute respiratory infections at the emergency room or hospital admission, this was not a major source of enrollment because many of these participants were not able to fulfill the longitudinal study requirements primarily due to large distances to our study site.

This study was funded by a National Institute of Allergy and Infectious Diseases (NIAID) Asthma and Allergic Diseases Cooperative Research Center (AADCRC) grant that also supports three related projects that will investigate key disease mechanisms, and utilize samples from this cohort. (1) The first project aims at identifying the molecular basis for RSV strain differences associated with mild and severe RSV respiratory infection. To do this, collaborators at Emory University School of Medicine (MLM, LJA) and J. Craig Venter Institute (SRD) will genotype RSV isolates of the INSPIRE cohort in order to identify RSV genotypes associated with bronchiolitis severity, childhood wheeze, and the development of asthma. (2) A second project will study the role of PGI_2_ in a mouse model of respiratory syncytial virus (RSV) infection (RSP). The ultimate goal is to determine if targeted therapies of the host immune response could prevent or treat severe RSV bronchiolitis and the subsequent development of asthma. (3) A third project is the longitudinal characterization of the infant and early childhood microbiome during health and acute respiratory illness, and the association of microbial diversity with both infant acute respiratory illness severity and childhood wheezing outcomes (TVH, CRS, and SRD).

In summary, this ongoing study will answer important questions about the mechanisms through which RSV causes acute and long term respiratory morbidity. This methodology paper outlines the study questions addressed, how the birth cohort (INSPIRE) was designed to address these questions, and provides preliminary enrollment data. Our unique study design targets infants born from June to December, such that they are on average 6 months of age during RSV circulation. With well-characterized respiratory infections during infancy, we are poised to address important questions about the causal role of RSV infection in the development of childhood wheezing and early childhood asthma. In addition to a carefully phenotyped cohort, we have a rich biospecimen repository. Our future plans include following this cohort to childhood asthma outcomes at age 6, characterizing the host responses to infection which are associated with protection from or risk of asthma, and understanding other associated risk factors including host genetics.

## Abbreviations

ARI: Acute Respiratory Infection
INSPIRE: Infant Susceptibility to Pulmonary Infections and Asthma Following RSV Exposure
LRI: Lower Respiratory Tract Infection
OR: Odds Ratio
URI: Upper Respiratory Tract Infection
PCRC: Pediatric Clinical Research Center
PBMC: Peripheral Blood Mononuclear Cells
RSV: Respiratory Syncytial Virus
